# Possible contribution of rare alleles of human ACE2 in the emergence of SARS-CoV-2 variants escaping the immune response

**DOI:** 10.3389/fimmu.2023.1252367

**Published:** 2023-10-10

**Authors:** Christian A. Devaux, Jacques Fantini

**Affiliations:** ^1^ Institut National des Sciences Biologiques (INSB), Centre National de la Recherche Scientifique (CNRS), Marseille, France; ^2^ Microbes, Evolution, Phylogénie et Infections, Faculté de Pharmacie, Aix Marseille Université, Marseille, France; ^3^ Institut National de la Santé et de la Recherche Médicale (INSERM) U_1072, Faculté des Sciences, Aix-Marseille Université, Marseille, France

**Keywords:** SARS-CoV-2, immune system, selection pressure, ACE2 receptor, genetic drift

## Abstract

Since the start of the SARS-CoV-2 pandemic, the rapid replacement of one lineage by another has been observed. Indeed, SARS-CoV-2 is evolving through a quasispecies mechanism leading to post-infection mutation selection under positive evolutionary pressure (host-driven viral evolution). These mutations may reduce the effectiveness of the specific neutralizing immune response against the virus. We provide here evidence that apart from the selection of SARS-CoV-2 variants by the immune system, selection by the cellular receptor can just as well select variants which escape neutralization.

## Introduction

It has always been considered that the immune response of the host (e.g.; neutralizing antibodies) is a major factor in positive selection of variant viruses (1, 2). In a human population, the encounter with a virus such as SARS-CoV-2 either induces a primary immune response or recalls preexisting memory immune cells specific for each individual histories of infections (3). Although intra-host analysis of SARS-CoV-2 evolution by deep sequencing methods has revealed the existence of one master mutant and numerous minor mutants in quasispecies, minor mutants may obtain a fitness advantage and become the master mutants under high selective pressure, ultimately favoring immune evasion (4). Intra-host genetic diversity was reported for patients treated with monoclonal antibodies targeting the viral spike, this therapeutic use of monoclonal antibodies leading to the selection of SARS-CoV-2 spike protein mutants less susceptible to this therapy. Cases of emergence of SARS-CoV-2 variants with an E_484_K substitution in their spike were reported after Bamlanivimab (5–7) and Bamlanivimab/Etesevimab monoclonal antibodies therapy (8). Notably, the genetic diversity of SARS-CoV-2 appears to be higher in immunocompromized chronically infected patients. A case report described the genetic evolution of SARS-CoV-2 in a 45-year-old immunocompromized male patient who had received antiviral treatment and anti-spike monoclonal antibodies. During the 151 days the patient had shed SARS-CoV-2, 45 genetic events were observed, including 24 non-synonymous mutations and 34 deletions. Twelve mutations were found in the spike among which substitutions E_484_K and N_501_Y (9). A similar observation was reported for a 59-year-old male patient immunocompromized due to follicular lymphoma and chronically infected with SARS-CoV-2 for 222 days. The authors documented the progressive emergence of viruses with critical spike mutations Q_493_K and N_501_T as majority quasispecies (10). The study of organ-specific intra-sample diversity using tissue samples collected post-mortem in March 2020 from 13 immunocompromized patients died from COVID-19, identified a N_501_Y in the spike of SARS-CoV-2, long before this mutation emerged worldwide (11).

However, another question quickly arose: could not the affinity of the virus for its receptor also play a very important role in the genetic drift of SARS-CoV-2 leading to an immune escape of variant viruses? After electrostatic interactions with lipid rafts (12), the first contact between the virus spike and the host is the virus cellular receptor. Therefore, it is likely that selection pressure impacting viral evolution relates to the virus’s affinity for its receptor. In the months following the announcement of the first cases of SARS-CoV-2 in China, angiotensin I converting enzyme 2 (ACE2) was identified as the receptor for this new virus (13) It was subsequently demonstrated that, in addition to serving as attachment receptor to the virus, the ACE2 molecule plays a crucial role in the pathophysiology of COVID-19 (14, 15).

## Role of ACE2 in the genetic drift of SARS-CoV-2

Since the World Health Organization declared SARS-CoV-2 to be a pandemic virus, the international community has endeavored to monitor the evolution of these viruses, new variants of which are regularly discovered. This genetic drift of SARS-CoV-2 is associated with the fact that the viral polymerase is error-prone, leading to replacements of SARS-CoV-2 lineages (16). In addition to viral enzyme-driven mutations, there are host parameters such as the apolipoprotein B mRNA editing enzymes (APOBEC) and/or adenosine deaminase acting on RNA (ADAR) systems (17, 18), a selection pressure by the immune system, and resistance factors to viral infections, which contribute to viral genomes evolution (19).

Until recently, the cellular receptor responsible for virus attachment was not considered to contribute to the selection of best-fit viruses in viral quasispecies. It was more generally accepted that the selection pressure exerted on the virus by neutralizing antibodies was responsible for most if not all mutations in the viral spike, and that in some cases these mutations could increase the affinity of the spike for ACE2. The in-depth study of SARS-CoV-2 sequences circulating in mink advanced the understanding of this complex molecular crosstalk, when it was demonstrated that SARS-CoV-2 present in mink farms had the propensity to evolve by missense mutations, some of which concerned the spike of SARS-CoV-2, such as the Y_453_F mutation (20). Using a structural biology approach, we demonstrated that this host-specific mutation gave a selective advantage to the virus for replication in mink (21). When this virus passes from mink to humans, the mutation is preserved but it is neutral in the binding to human ACE2, inducing neither selective advantage nor an unfavorable effect. SARS-CoV-2 circulating in deer also adapts by mutations that result in viral spikes with F_486_L and N_501_T substitutions that are also host-specific. During the outbreak of SARS-CoV-2 in hamsters, a mutation D_427_G was identified and we demonstrated that it annihilated the torsion that pushes the Q_34_ amino acid of the hamster ACE2 in unfavorable direction for interaction with the viral spike. This D_427_G substitution could give a selective advantage to this virus to interact with the human ACE2 in case of transmission from hamster to human (22). Altogether, these results indicate that the inter-species polymorphism of ACE2 is an important factor in the selection of new variants.

The pressing question then arose of the role of human ACE2 polymorphism in the emergence of SARS-CoV-2 variants. Thanks to the human genome project, we now know that ACE2 is a polymorphic molecule. Certain alleles more or less frequent dominate in regions of the world and are absent elsewhere (23). Considering the N_501_Y mutation in the spike, we wondered about the existence of ACE2 alleles that could promote its emergence in the human population by intra-specific transmission of the virus. Very recently, we obtained evidence indicating that an N_501_Y variant could be selected when SARS-CoV-2 with an N_501_ was transmitted to people with a rare ACE2 allele (MAF=0.02%) expressing an E_329_G, found in the European human population and absent in Asian people. When hundreds of millions of people are infected, the probability that a SARS-CoV-2 virus will encounter a host that expresses this rare allele is not negligible. Notably, the N_501_Y variant of concern (B1.1.7), emerged in the UK (24). Very recently, we reported structural evidence that the E_329_G substitution in ACE2 is much more favorable to interaction with the mutated spike protein N_501_Y than it is with E_329_, a physical contact (hydrogen bond) being established between N_501_Y and Q_325_ for ACE2 with E_329_G, whereas this contact does not appear for ACE2 with E_329_ (25).

## Discussion

By studying the dynamics of emergence of the N_501_Y lineages of SARS-CoV-2 during the COVID-19 pandemic, we found evidence that a cellular receptor-driven selection process can occur during human-to-human transmission of viruses carrying an asparagine at amino acid position 501 (N_501_) in their spike protein. More precisely, by molecular modeling we demonstrated for the first time, that the encounter of an N_501_ virus with a rare allele of ACE2 (E_329_G) could lead to the selection of an N_501_Y lineage SARS-CoV-2 viruses exhibiting higher affinity for the receptor (25) and lower susceptibility to neutralization by antibodies (26, 27).

**Figure 1:**
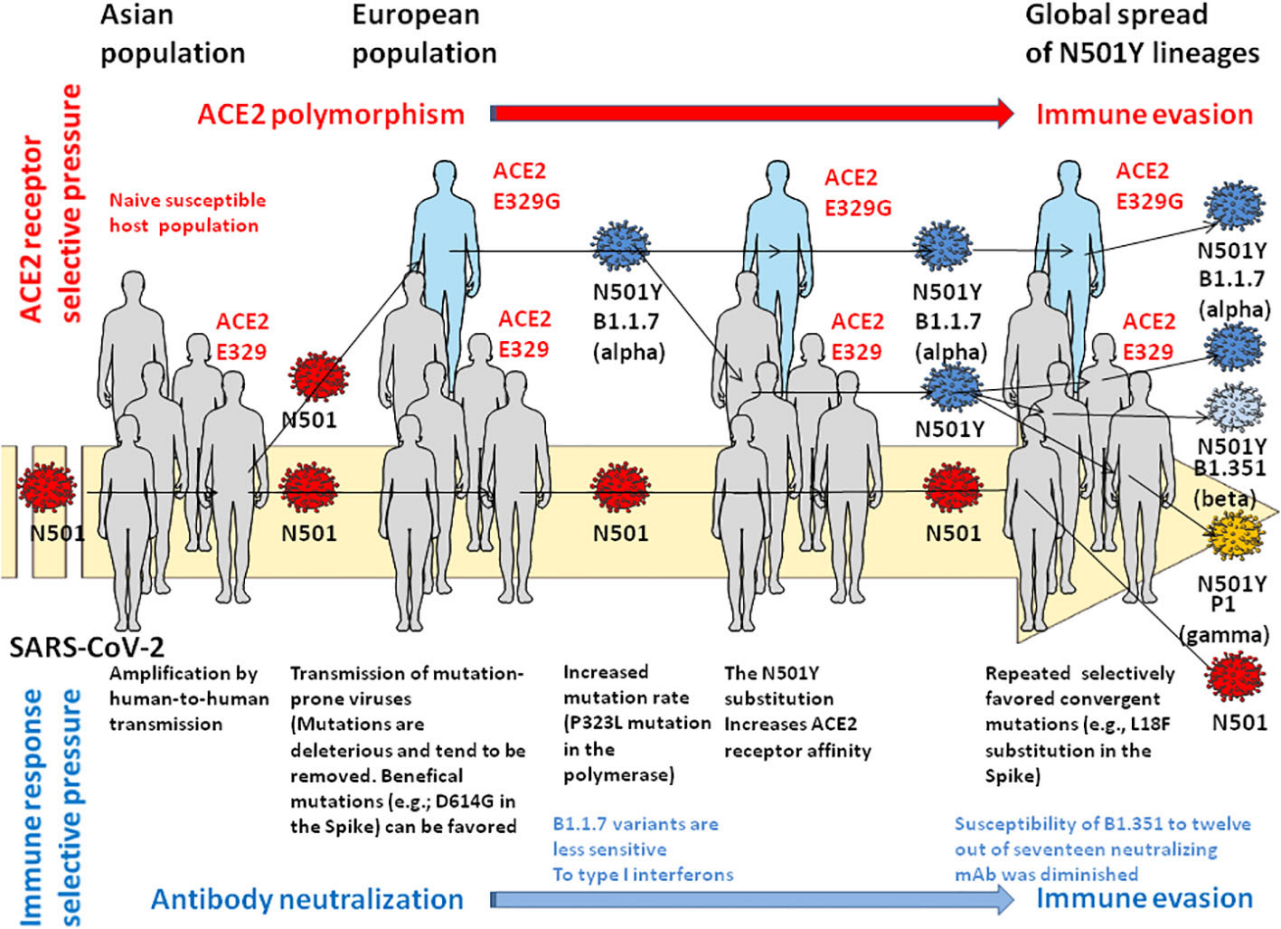
Hypothetical model of immune escape by selection pressure at the level of the viral receptor. This model proposes the intervention of an individual expressing a rare allele of ACE2 (E_329_G) in the selection of the N_501_Y lineage of SARS-CoV-2 then its transmission to human populations expressing the major ACE2 (E_329_) allele. In this model, three main forces act on the genetic drift of the virus: the fitness capacity of the virus, the sequence of the host’s ACE2 serving as a receptor for the virus and, the host’s anti-SARS-CoV-2 immune response. For more detail on the genetic drift of the virus see reference (17) and reference (18) for the immune response aspect. Please refer to the main text and reference (25) for descriptions of variants of concern.

Of course, we have no formal proof that the history of the emergence of N_501_Y viruses occurred through ACE2 rare allele selection in humans. Alongside the selective advantage provided by the N_501_Y substitution for the replication of SARS-CoV-2 in humans, we know that N_501_Y also gives a selective advantage to the virus for replication in mink, mice and deer which could also have contributed to the emergence of such a variant through inter-species transmission (e.g., there are Q or G, Q, and D amino acids in place of the E at position 329 in the ACE2 sequences of hamster, mice, and deer, respectively). However, the model of virus evolution by selection in a human host expressing a rare ACE2 allele (**Figure 1**), followed by a boomerang effect in the general population, remains a very attractive hypothesis. In the study by Van cleemput and colleagues (11), it would have been interesting to know the ACE2 allele carried by the immunocompromized patients whose viruses, found in samples from March 2020, carry the N_501_Y mutation in their spike. Since the Alpha (B1.1.7; first detection was in November 2020 in UK), Beta (B.1.351; first detection was in October 2020 in South Africa) and Gamma (P.1; first detection was in January 2021 in Japan) variants of SARS-CoV-2 that carried this N_501_Y substitutions appeared during late 2020 and early 2021, it remains possible that the N_501_Y lineage first emerged in an immunocompromized male patient infected with SARS-CoV-2 who expressed a rare allele of ACE2. The fact that the *ACE2* gene is on chromosome X (28, 29) supports this hypothesis. However, more than three years after its emergence, it would be extremely complex to attempt to trace back the hypothetical origin of the N_501_Y variant of concern in a population expressing the rare allele E_329_G of ACE2 in the UK, by direct genotyping. As suggested by a reviewer of this paper, an elegant way to approach the problem today might be to carry out an *in vitro* experiment of serial passages of an ancestral SARS-CoV-2 that does not contain the N_501_Y in a compatible cell line expressing an ACE2 with E_329_G substitution in order to observe the possible emergence of a N_501_Y variant. We are not discussing it in this short communication, but the N_501_Y variant of concern could also have been selected for adaptation to another receptor or co-receptors (e;g., NRP-1) (30), in different tissues and organs in humans (which could also be regarded as an example of host receptor-driven virus mutation). If the virus receptor expressed by the host can play a selection role in the same way as the immune system, in an immunocompromized patient the role of the receptor can theoretically become preponderant. Moreover, this model of virus selection by adaptation to the receptor could be interesting to explore for other mutations that affect the Spike protein of SARS-CoV-2.

Finally, although the accumulation of whole genome sequences with SARS-CoV-2 has made it possible to observe these rare events, it is likely that this selection mechanism may exist for other viruses and their respective receptors. This opens up an avenue for the exploration of a new mechanism of virus evolution. It is time to take into account both the selection pressure of the immune system and that imposed by the cellular receptors of viruses in their genetic drift.

## Data availability statement

The original contributions presented in the study are included in the article/supplementary materials. Further inquiries can be directed to the corresponding author.

## Author contributions

All authors listed have made a substantial, direct, and intellectual contribution to the work and approved it for publication. All authors contributed to the article and approved the submitted version.
